# Development of *oriC*-plasmids for use in *Mycoplasma hyorhinis*

**DOI:** 10.1038/s41598-017-10519-3

**Published:** 2017-09-06

**Authors:** Hassan Z. A. Ishag, Qiyan Xiong, Maojun Liu, Zhixin Feng, Guoqing Shao

**Affiliations:** 1 Institute of Veterinary Medicine, Jiangsu Academy of Agricultural Sciences, Key Laboratory of Veterinary Biological Engineering and Technology, Ministry of Agriculture, National Research Center for Engineering and Technology of Veterinary Bio-products, Nanjing, 210014 China; 2 College of Veterinary Science, University of Nyala, Nyala, Sudan

## Abstract

*Mycoplasma hyorhinis* (*M*. *hyorhinis*) is an opportunistic pig pathogen, belonging to the class Mollicutes. It causes polyserositis, arthritis and cancers *in vitro*, increasing attention of the researchers. Currently, there is no available genetic tool to manipulate its genome. This study describes a development of *oriC*-plasmids harboring either large (*pGEMT-LoriC*) or minimum (*pGEMT-MoriC*) origin of replication (*oriC*) of *M*. *hyorhinis* along with tetracycline resistance marker.These plasmids were successfully transformed into *M*. *hyorhinis* with average transformation frequency of 1.5 × 10^−4^ and 2.0 × 10^−5^ transformants/CFU for *pGEMT-LoriC* and *pGEMT-MoriC* respectively, and were integrated at the chromosomal *oriC* as well as remained freely replicating. We also constructed a *Mini-oriC-HT1* targeting plasmid by inclusion of *hlyC* arms and was used to inactivate *hlyC* at average frequency of 50%. The efficiency of *hlyC* inactivation was further improved (by 90%) when *Mini-oriC-HT2* that contains *E*. *coli recA* was used. In both cases, hemolysin mutant bacteria diminished the ability to lyse mouse RBCs compared to wild-type (*P* < *0*.*001*). *OriC*-plasmids described in this study may, therefore open the way for functional genomics in *M*. *hyorhinis*. Furthermore, this is a first study demonstrated the gene associated with a hemolytic phenotype in mycoplasmas.

## Introduction

*Mycoplasma hyorhinis* (*M*. *hyorhinis*), is an opportunistic pathogen of pigs associating with respiratory tract infections and arthritis^1^. *M*. *hyorhinis* is most often detected as contaminants in cell cultures^2^. In addition, chronic *M*. *hyorhinis* infection has been found to play a role in the development of several types of cancers *in vitro* ^2, 3^, increasing attention of the researchers.

The genome sequence of *M*. *hyorhinis* has been completed and published several years ago^4^. However, studies to manipulate the genome of *M*. *hyorhinis* and understand the molecular pathogenesis suffers from the lack of genetic tools available for the pathogen.

For many mycoplasmas including *M*. *hyorhinis*, the transposon mutagenesis and its derivatives have been developed for genetic studies^5, 6^. However, random integration of the transposon into the chromosome did not allow the specific gene targeting analysis.

Suicide plasmids have also been used in Mollicutes to inactivate targeted genes via homologous recombination between the gene cloned in the plasmid and its homologues sequence in the host genome. Because of low recombination frequency that may cause by the low transformation efficiency, these vectors were applied only in very few mycoplasmas including *Mycoplasma mycoides* subsp. *capri* ^7^, *Mycoplasma gallisepticum* ^8^, *Acholeplasma laidlawii* ^9^ and *Mycoplasma genitalium* ^10, 11^. Indeed, in our laboratory, we have developed suicide plasmids and have been successfully used to inactivate *hlyC* in *M*. *hyorhinis* ^12^.

Recently, replicative plasmids have been established for a number of Mollicutes including *Spiroplasma citri* ^13^, *Mycoplasma pulmonis* ^14^, *Mycoplasma mycoides* subsp. *capri*, *M*. *mycoides* subsp. *Mycoides small-colony type* ^15^, *Mycoplasma capricolum* subsp. *capricolum* ^15, 16^, *Mycoplasma agalactiae* ^17^ and *Mycoplasma gallisepticum* ^18^. The typical *oriC*-plasmid contains an origin of replication (*oriC*) along with tetracycline resistance marker and sufficient sequence homologous to chromosomal DNA^6^ to insert into the chromosome of the organism via homologous recombination. These *oriC*-plasmids have been used in some studies to disrupt targeted genes, complement mutants^15, 16, 19^ and also to express foreign genes^20^.

More recently, in-Yeast engineering of *Mycoplasma mycoides* subsp. *capri GM12* (Mmc) genome using CRISPR/Cas9 system, has also been established and was used to disrupt mycoplasma glycerol-3-phosphate oxidase-encoding gene (*glpO*)^21^. However, cloning and assembly of a synthetic bacterial genome in yeast followed by a back transplantation into recipient bacterial cells is a challenge.

At present, the *oriC*-plasmids have not been studied in *M*. *hyorhinis*. The aim of this study was to construct and evaluate the usefulness of these plasmids as genetic tools in *M*. *hyorhinis*. Here, the *oriC*-region of *M*. *hyorhinis* was characterized and used for the development of *oriC*-plasmids that replicates in *M*. *hyorhinis*.

In many cases, the *oriC*-plasmids harboring large *oriC*-fragments tend to integrate into the *oriC*-region of the genomic DNA by homologous recombination between the gene fragment cloned on the plasmid and the corresponding site on the chromosome due to the sequence homology. The stability of the *oriC*-plasmids can be improved by utilizing a minimum *oriC* that reduces the possibility of the integration of the plasmid into the host chromosome^14, 18, 22^. Herein, a mini-*oriC* plasmid that could replicate and maintained stably extra-chromosomally has been developed and evaluated. This study also provides evidence of homologous recombination occurring in *M*. *hyorhinis*.

The hemolytic activity has been described in other mycoplasmas^23^. Since the HUB-1 genome sequence has a *hlyC* (NCBI Reference Sequence: WP_014582557.1) related sequence identified by automated prediction, it is hypothesized that this sequence is involved in hemolysis. Therefore, *hlyC* was chosen as the target gene for these experiments. We initially confirmed that, the supernatant of wild-type *M*. *hyorhinis* can lyse mouse RBCs (Supplementary Fig. S1). When the mini-*oriC* plasmid that contained relatively small regions (~500 bp) of homology with chromosomal DNA of *hlyC* flanking *tetM* used to transform *M*. *hyorhinis*, the *tetM* inserted into the chromosome and disrupted the *hlyC*. The supernatant of the hemolysin mutant strain diminished the ability to lyse mouse RBCs. Therefore, this manuscript demonstrates that *hlyC* may be involved in mouse RBC lysis.

*RecA* plays an important role in DNA recombination and repair^24^. In *M*. *mycoides* subsp. *capri*, the efficiency of homologous recombination using suicide plasmids was enhanced by the inclusion of a *recA* (from *E*. *coli*) into the constructs^7, 25^. Therefore, in this study, we further augmented the occurrence of the homologous recombination at the *hlyC* site by introducing *recA* into our targeting *oriC*-plasmids.

The ability to transform *M*. *hyorhinis* has been long described. However, the transformation procedure and the *oriC*-plasmid systems developed in this study, have the potential to be used in targeted gene disruption, and may also in gene complementation and expression studies in this organism and possibly other mycoplasmas.

## Results

### Vectors construction

**Figure 1:**
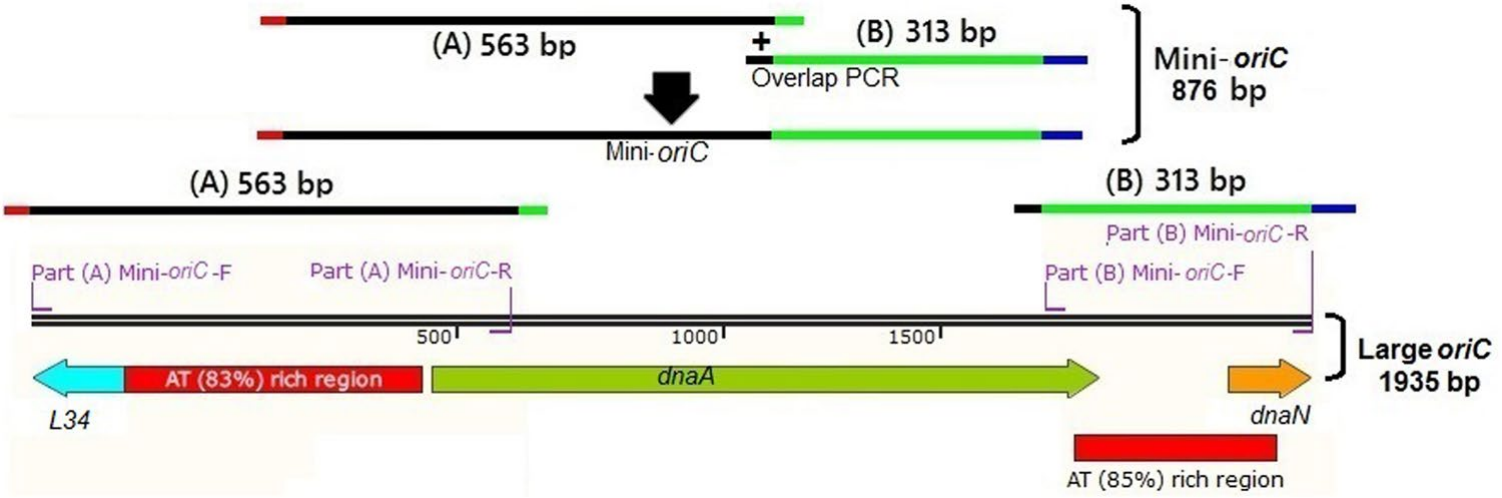
Prediction of the origin of replication of *M*. *hyorhinis*. A 1935-bp large *oriC* region (L*oriC*) of *M*. *hyorhinis* strain HUB-1 was predicted around the *dnaA*. It contains short AT-rich regions of 83% and 85% upstream and downstream *dnaA* respectively. To generate mini-*oriC*, the regions upstream and downstream *dnaA*, were amplified as fragment A (563-bp) and fragment B (313-bp). These two fragments were joined by overlapping PCR to form mini-*oriC* (M*oriC*, 876-bp).

No functional *oriC* had previously been isolated from *M*. *hyorhinis*. The large *oriC* region (L*oriC*) of *M*. *hyorhinis* (1935-bp) which consisted of the complete *dnaA* and intergenic regions upstream and downstream of the *dnaA* (NCBI Reference Sequence: WP_020104468.1) were identified (Fig. 1). The genomic regions upstream of the *dnaA* contained a single putative DnaA box and the AT-rich region is about 83% (Fig. 1). Likewise, the genomic regions downstream of the *dnaA* contained a single putative DnaA box and the AT-rich region is about 85% (Fig. 1). The minimum *oriC* region (M*oriC*) of about 876-bp, only contains the regions upstream and downstream the *dnaA* (Fig. 1).

**Figure 2:**
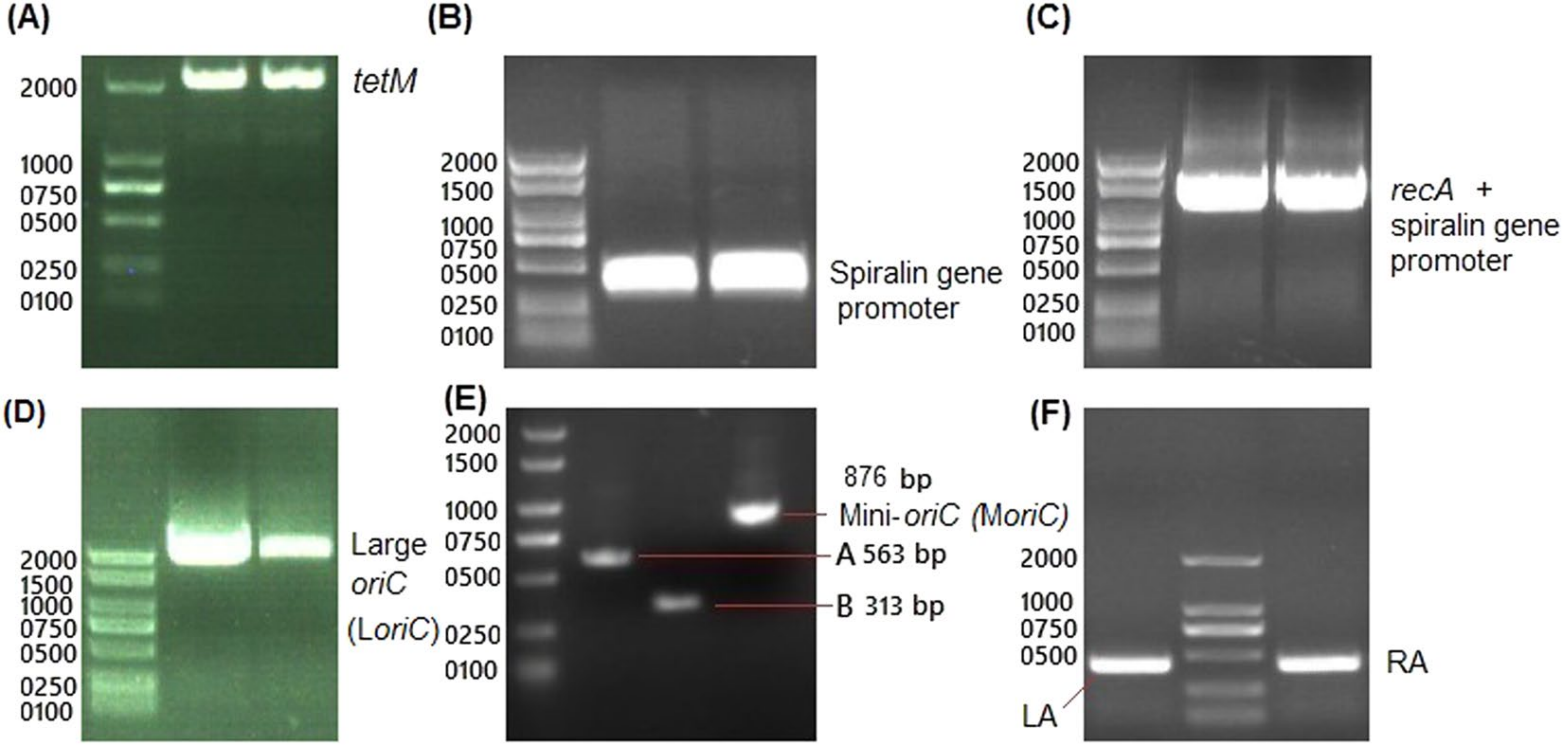
PCR products of fragments required to construct *oriC*-plasmids for *M*. *hyorhinis*. The *tetM* 1917-bp (**A**), spiralin gene promoter 313-bp (**B**), spiralin gene promoter joined with *recA* to form a single fragment of about 1375-bp (**C**), large *oriC* (L*oriC*) 1935-bp (**D**), mini-*oriC* (M*oriC*) 876-bp (**E**) and hemolysin left arm (LA) 437-bp and right arm (RA) 405-bp (**F**) were shown. The gels images were cropped and full-length gels are included in the Supplementary Fig. S8.

**Figure 3:**
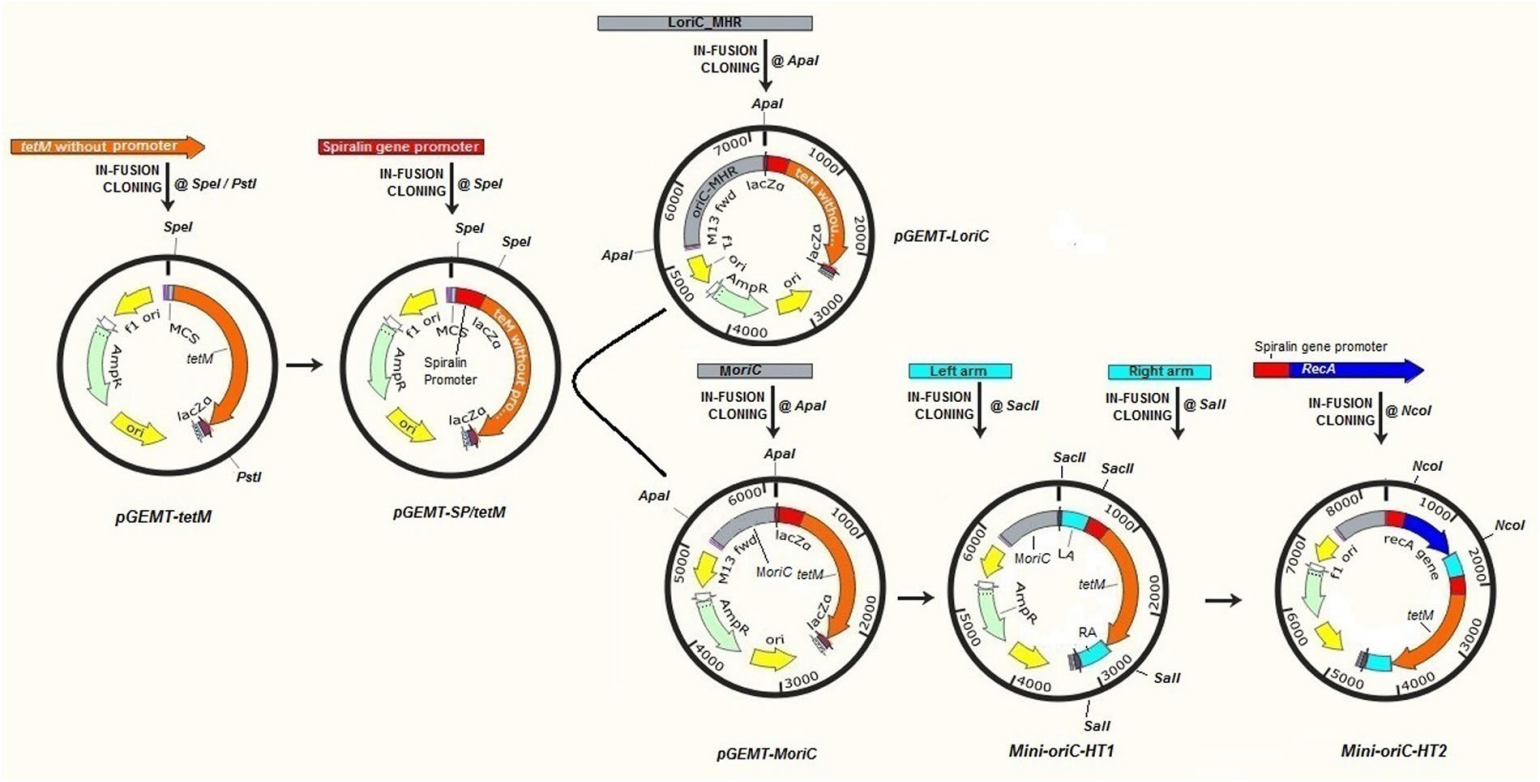
Schematic representation of the procedures for the construction of *oriC*-plasmids for *M*. *hyorhinis*. The *tetM* was cloned at *Pst*I/*Spe*I restriction enzymes sites, spiralin gene promoter was cloned at *Spe*I restriction enzyme site, L*oriC* and M*oriC* were cloned at *Apa*I restriction enzyme site, Hemolysin left arm (LA) was cloned at *Spe*I restriction enzyme site, Hemolysin right arm (RA) was cloned at *Sal*I restriction enzyme site and finally, the *recA* spliced with spiralin gene promoter was cloned at *Nco*I restriction enzyme site. The resultant *oriC*-plasmids along with their vectors designations were presented.

The PCR products of *tetM* 1917-bp (without promoter), spiralin gene promoter 313-bp, large *oriC* (L*oriC*) 1935-bp, mini-*oriC* (M*oriC*) 876-bp, *recA* 1062-bp, hemolysin left arm (LA) 437-bp and hemolysin right arm (RA) 405-bp were prepared (Fig. 2) and separately cloned into *pGEM®-T* vector. To determine whether the L*oriC* region is able to function as an autonomously replicating sequence, we first constructed *pGEMT-LoriC* plasmid, harboring L*oriC*, and later reduced the *oriC* to a minimum and cloned to generate *pGEMT-MoriC*. To target *hlyC*, both hemolysin arms were cloned flanking the *tetM* to yield *Mini-oriC-HT1* plasmid. Finally, the efficiency of hemolysin targeting had improved by introducing *recA* expressed by spiralin gene promoter to yield *Mini-oriC-HT2* plasmid. The general strategy used to construct the *oriC*-plasmids was shown in (Fig. 3).

### Transformation of *M*. *hyorhinis*

Following transformation with *pGEMT-LoriC* and *pGEMT-MoriC* plasmids, tetracycline resistant colonies appeared within 3 to 5 days on KM2-agar plates containing 2.5 µg/ml tetracycline. During the indicated time period, no spontaneous tetracycline-resistant colonies appeared for untransformed controls. The presence of the plasmids in the transformants was confirmed when *tetM* (part of the plasmid) was detected by *tetM* specific PCR (with **P9** primers, Table 1) using DNA template prepared from sub-cultured colonies (Supplementary Fig. S2). The transformation frequency of *pGEMT-LoriC* plasmid was 1.5 × 10^−4^ transformants per colony-forming unit (CFU) compared to 2.0 × 10^−5^ transformants per CFU for *pGEMT-MoriC* plasmid.

**Table 1:** List of primers and their sequences used in this study.

Primer information	Primer sequence (5′–3′)	Cloning site
P1 (1917-bp): *tetM* (no promoter)	F: GAAATATAAGAAACTAGTATGAAAATTATTAATATTGGAGTTTTAGCTCATGTTGATGC	*Spe*I *Pst*I
	R: TTCGATTGGTCGACCTGCAGTTATTTTATTGAACATATATCGTACTTTATCTATCCG	
P2 (313-bp): (Spiralin gene promoter)	F: GAGCATGCGACGTCGATCCTCCTAAAGCAGAATATCCGTTTGAA	*Spe*I
	R: TAATTTTCATACTAGTTTCTTATATTTCCTTTCTCTATTAAGTAGTGTTTTTATTAAAAGC	
P3 (1935-bp): (L*oriC*)	F: CTATAGGGCGAATTGGGCCCTACCTTTTGCTCTTCTTGCTGCTAAAACT	*Apa*I
	R: GAGCATGCGACGTCGATCCTCCTAAAGCAGAATATCCGTTTGAA	
P4A (563-bp): (M*oriC*)	F: CTATAGGGCGAATTGGGCCTACCTTTTGCTCTTCTTGCTGCTAAAACTTT	*Apa*I
	R: GCATACTTAATCGCAGGTTCTTTCTGCTTTTGGAAATTAACTTCATCTGA	
P4B (313-bp): (M*oriC*)	F: TCAGATGAAGTTAATTTCCAAAAGCAGAAAGAACCTGCGATTAAGTATGC	*Sac*II
	R: GAGCATGCGACGTCGATCCTCCTAAAGCAGAATATCCGTTTGAA	
P5 (437-bp): (Hemolysin-LA)	F: GCGGGATATCACTAGTCCAGGCGCACTTACAAAAGATCAC	
	R: CTTCACTGTTTTCTTGTTCACTAACATCACAATATCAGCATCTTGCTCGATAG	
P6 (405-bp): (Hemolysin-RA)	F: ATAACTGCAGGTCGACAATCGAAGCTTGATTAGAACATCATAGC	*Sal*I
	R: CTCCCATATGGTCGACTACTACAATTACTGCTTTCCGAGTTATTAAAATAC	
P7 (1062-bp): (*RecA*- splicing-Spiralin gene promoter)	F: GAAAGGAAATATAAGAAATGGCTATCGACGAAAACAAACAG	*Nco*I
	R: TATCCCGCGGCCATGGTTAAAAATCTTCGTTAGTTTCTGCTACGCCT	
P8 (313-bp): (Spiralin gene promoter-splicing-*recA*)	F: CTCCCGGCCGCCATGTTAGTGAACAAGAAAACAGTGAAGCACCAG	
	R: TTTCGTCGATAGCCATTTCTTATATTTCCTTTCTCTATTAAGTAGTGTTTTTATTAAAAG	
P9 (339-bp): (*tetM* screening)	F: GCAGTTATGGAAGGGATACG	—
	R: TTCTTGAATACACCGAGCAG	
P10 (4037-bp): (Integration predicted product)	F: CAGCATTGACAAATTTTTTCGGAATCGAG	—
	R: CGTTTCCCTCTATTACCGTATCCCATTG	
P11 (1566-bp for Wild-type and 3072-bp for the mutant): (*hylC* flanking and sequencing)	F: CAATTAGCACGTGAATTAGACACACCG	—
	R: CCATAATTAGCCTTCATTTTTCTTTGTGATTTGAATTTC	
P12 (single crossover)	F: GAGTCAAGGTTTTCAAGGATTCCAGTTAG	—
	R: GCCCTGTTAGTACCCCAGCAGATTTTC	

Primer sequences are shown along with the size of the PCR product generated. F = forward, R = reverse, P = primer, LoriC = Large origin of replication, MoriC = minimum origin of replication, tetM = tetracycline hydrochloride, LA = left arm, RA = right arm and recA = recombinase gene A.

### Analysis of homologous recombination at the *oriC* site of *M*.*hyorhinis* genome

**Figure 4:**
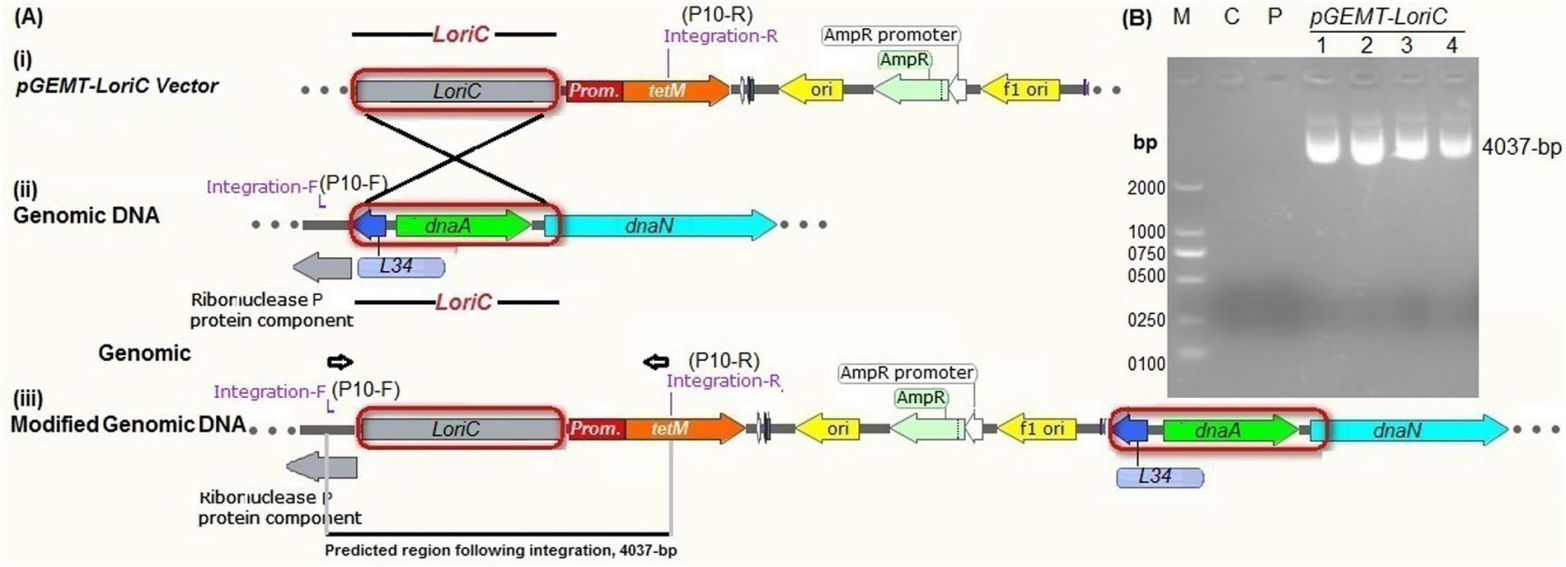
Prediction of the possible integration of *pGEMT-LoriC* plasmid containing large *oriC* (L*oriC*) at the *oriC*-region of *M*. *hyorhinis* (**A**). Following integration at the *oriC* region, a fragment of about 4037-bp could be detected with PCR using integration primers (**P10**, Table 1): forward integration primer (**P10-F**, Table 1) binds specifically to *M*. *hyorhinis* genome upstream the L*oriC*, while reverse integration primer (**P10-R**, Table 1) binds specifically to *tetM* of the *pGEMT-LoriC* plasmid (**B**) to yield a product of predicted size of 4037 bp. The gel image was cropped and full-length gel is included in the Supplementary Fig. S9.

The *oriC*-plasmids containing L*oriC* fragment, have been shown to integrate into the host chromosome via homologous recombination due to the sequence homology with *oriC*-region. Here, we evaluated whether our *pGEMT-LoriC* had integrated into the *M*. *hyorhinis* genome, and also investigated the site at which the integration had occurred. Briefly, *M*. *hyorhinis* DNA extracted from transformed (at 5^th^ passages) and untransformed control cultures, were subjected to the PCR analysis. Based on *M*. *hyorhinis* genomic sequence and *pGEMT-LoriC* plasmid, we designed integration primers (**P10**, Table 1) to investigate the predicted integration site of the plasmid at the *oriC*-region (Fig. 4A). The forward integration primer (**P10-F**, Table 1) was designed to specifically bind up-stream the L*oriC* at the *M*. *hyorhinis* genome and the reverse integration primer (**P10-R**, Table 1) was designed to specifically bind the *tetM* of the *pGEMT-LoriC* plasmid. Following plasmid integration by single cross-over at the *oriC*-region of *M*. *hyorhinis*, the predicted size of the PCR product was found to be 4037-bp (Fig. 4A). Indeed, the PCR product using integration primers (**P10**, Table 1) and DNA extracted from *pGEMT-LoriC* transformants, produced fragments of 4037-bp (Fig. 4B) indicating the integration of *pGEMT-LoriC* at the predicted *oriC* region. Analysis of the DNA extracted from *pGEMT-LoriC* transformants by *tetM* specific PCR (with **P9** primers, Table 1), had detected the *tetM* and further confirmed the presence of the plasmid (Supplementary Fig. S3). In contrast, if the plasmid replicating in an extrachromosomal form (did not integrate), then the PCR fail to generate a band due to the specificity of the specific integration primers (**P10**, Table 1) (because the forward integration primer binds specifically to *M*. *hyorhinis* genome upstream to L*oriC*, while reverse integration primer binds specifically to *tetM* of the *pGEMT-LoriC* plasmid). We also predicted the size of the PCR product when *pGEMT-MoriC* integrated at the *oriC*-site (2122-bp), where we get no PCR product (Supplementary Fig. S4), indicating the absence of plasmid integration at this region by single cross-over.

### Inactivation of *hlyC* using *Mini-oriC-HT1*

**Figure 5:**
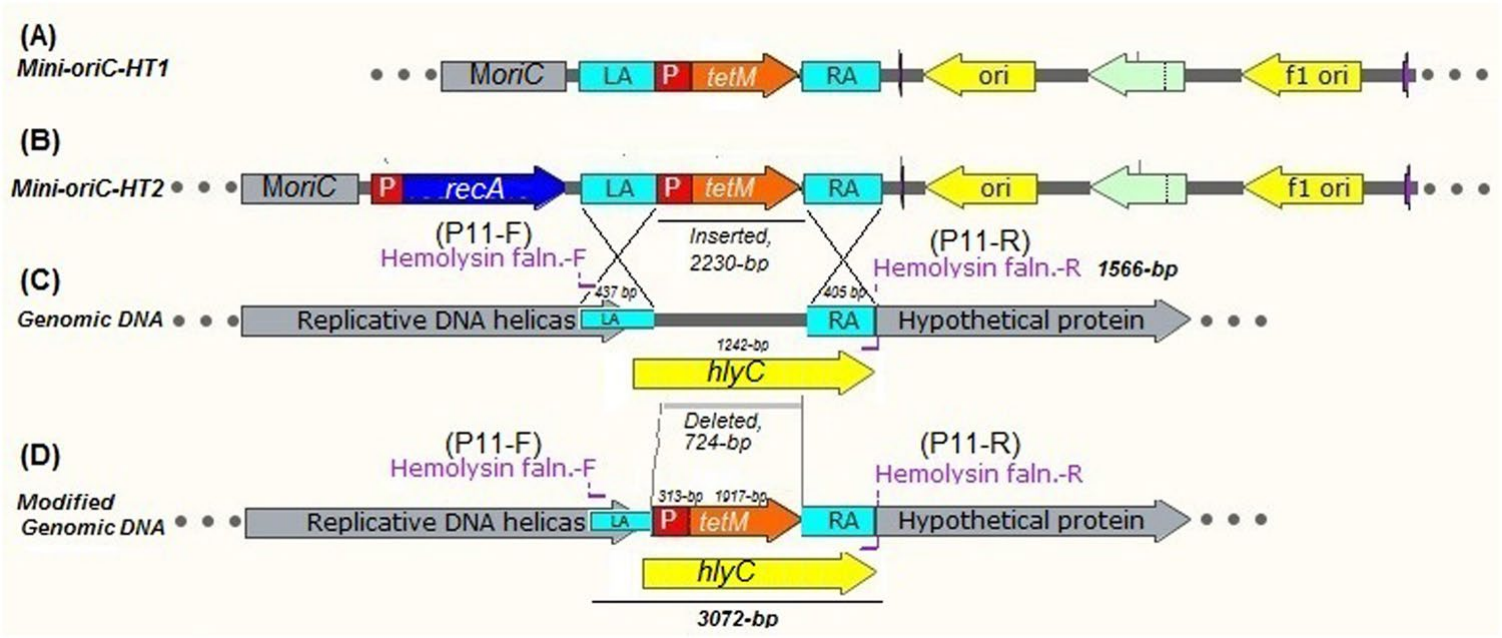
Prediction of the possible insertion of *tetM* along with spiralin gene promoter at the hemolysin site using *Mini-oriC-HT1* and *Mini-oriC-HT2* targeting plasmids. Following insertion at hemolysin site, a fragment of about 3072-bp could be amplified with PCR using *hlyC* flanking primers. Wild-type *hlyC* exhibits 1566-bp. LA = left arm, RA = right arm.

**Figure 6:**
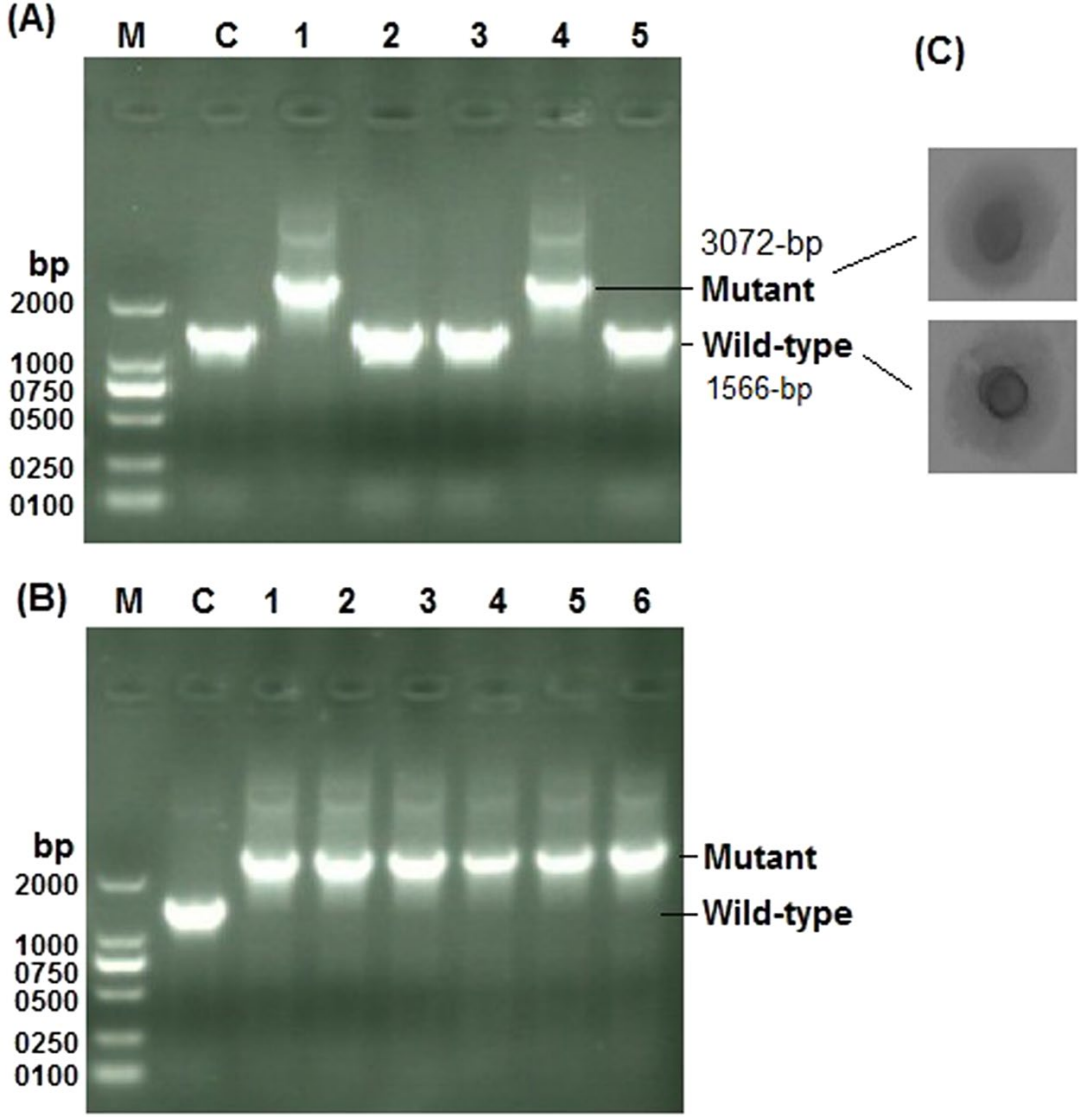
Analysis of *hlyC* disruption: DNA was extracted from the grown culture of *M*. *hyorhinis* transformed with *Mini-oriC-HT1* (**A**) and *Mini-oriC-HT2* (**B**) targeting plasmids along with control untransformed cultures, was subjected to PCR analysis using *hlyC* flanking primers (**P11**, Table 1) to investigate the integration of the *tetM* into *M*. *hyorhinis* genome at *hlyC* site. Wild-type hemolysin exhibits 1566-bp while mutant hemolysin that encodes *tetM* along with spiralin gene promoter has about 3072-bp. (**C**) the phenotype of the wild-type and mutant colonies of *M*. *hyorhinis*. The gel image was cropped and full-length gel is included in the Supplementary Fig. S10.

The hemolytic activity has been described in other mycoplasmas^23^. However, there is no specific gene in mycoplasmas identified to be associated with the hemolysis. In this study, we first confirmed that, the supernatant collected from *M*. *hyorhinis* can lyse mouse RBCs (Supplementary Fig. S1). Since the HUB-1 genome sequence encodes a *hlyC* related sequence determined by automated prediction, this sequence is hypothesized to be involved in hemolysis. Therefore, we chose to inactivate the *hlyC* as it’s easy to measure the phenotype of the mutant. In other mycoplasmas, the L*oriC*-fragment drives the plasmid to integrate at the *oriC*-region of the host due to sequence homology. Therefore, we constructed an *oriC*-plasmid that contains minimum *oriC*-region (876-bp) and was named *pGEMT-MoriC*. Since we aimed to evaluate whether *pGEMT-MoriC* plasmid could be used for *hlyC* inactivation. We cloned hemolysin left and right arms into the *pGEMT-MoriC* plasmid to flank *tetM* and the spiralin gene promoter and to yield *Mini-oriC-HT1* targeting plasmid. The resultant mutant strain expected to confer resistance to tetracycline when added to the medium at the appropriate concentration. The following transformation with *Mini-oriC-HT1* plasmid and plating in KM2-agar containing 2.5 µg/ml tetracycline, we observed the growth of tetracycline resistance colonies. Selected colonies sub-cultured in KM2 medium containing 5.0 µg/ml tetracycline and subjected to PCR analysis using *hlyC* flanking primers (**P11**, Table 1) located upstream and downstream of the *hlyC* respectively. The wild-type *hlyC* produces a predicted PCR product of about 1566-bp with these primers (Fig. 5) indicating the absence of *tetM* insertion at *hlyC* site. When *hlyC* is disrupted by inserting *tetM* along with spiralin gene promoter, the predicted sequence will increase (3072-bp) and could be PCR amplified with *hlyC* flanking primers (**P11**, Table 1) (Fig. 5). In our study, we observed a larger PCR product, presumably 3072-bp in size, following insertion of *tetM* and the spiralin gene promoter into *hlyC* via homologous recombination (Fig. 6A). Five colonies out of ten (50%) from two repeated experiments were produced with the predicted larger PCR product targeting *hlyC* when *Mini-oriC-HT1* was used. This indicates the success of the specific targeting and inactivation of the *hlyC*. It also indicates the occurrence of homologous recombination in *M*. *hyorhinis*.

### Improvement hemolysin targeted inactivation by the inclusion of *recA*

To improve the frequency of gene targeting, we inserted *recA* (from *E*. *coli*) controlled by the spiralin gene promoter into *Mini-oriC-HT1* to yield *Mini-oriC-HT2* targeting plasmid. The previous study indicated the usefulness of *recA* to enhance homologous recombination in *M*. *mycoides* subsp. *capri* ^7^. Similar results were observed with our suicide vectors^12^. Following transformation with *Mini-oriC-HT2* plasmid and sub-culture of 5 resistance colonies, we observed the predicted size of PCR product (3072-bp) in all the selected colonies indicating the insertion of *tetM* at the hemolysin site (Fig. 6B). Similar results were obtained when the experiment repeated. Overall, an about 9/10 (90%) hemolysin targeting efficiency from two repeated experiments was obtained when *recA* incorporated into the targeting *oriC*-plasmid (*Mini-oriC-HT2*). No significant change in the phenotype of the wild-type and mutant colonies was observed (Fig. 6C).

**Figure 7:**
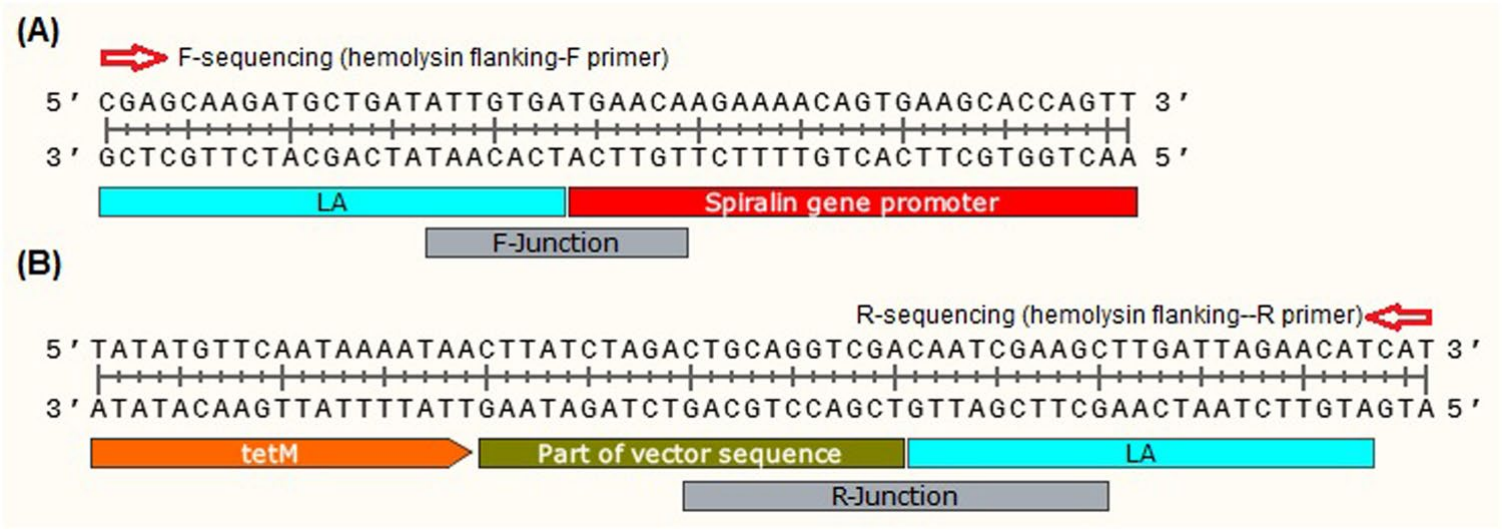
DNA sequencing analysis of the large DNA fragment (mutant *hlyC*, 3072-bp) amplified with *hlyC* flanking primers (**P11**, Table 1) from DNA of *M*. *hyorhinis* cells transformed with *Mini-oriC-HT1* (**A**) and *Mini-oriC-HT2* (**B**) targeting plasmids. The sequencing was performed in both forward and reverse directions using *hlyC* flanking primers, **P11** (Table 1)”

The *tetM* insertion at the hemolysin site was further investigated with *tetM* specific PCR (with **P9** primers, Table 1), using the 3072-bp PCR product amplified with *hlyC* flanking primers (**P11**, Table 1) as a template (Supplementary Fig. S5). The 3072-bp PCR product amplified with *hlyC* flanking primers was directly sequenced to analyze the junctions and the presence of *tetM* along with spiralin gene promoter. Analysis of the DNA sequencing results indicates the presence of sequences of *hlyC* arms, spiralin gene promoter as well as the *tetM* (Fig. 7), confirming the insertion of *tetM* along with the spiralin gene promoter at *hlyC* site.

Further, we predicted the occurrence of single cross-over between the plasmid and any arm of *hlyC* leading to the integration of the full *Mini-oriC-HT1* plasmid that would result in PCR amplification of *tetM* thus generating positive PCR results. The phenotype of such transformants should have a hemolysin positive phenotype. To investigate this event, we predicted the integration of the *Mini-oriC-HT1* plasmid by a single cross over at the hemolysin right arm site. We designed single cross-over forward and reverse primers (**P12**, Table 1). The single-cross forward primer (**P12-F**, Table 1) binds the upstream region of hemolysin right arm (absent if double-cross over had occurred) while the single-cross reverse primer (**P12-R**, Table 1) binds the *tetM* present in the *Mini-oriC-HT1* plasmid. In our experiment, we failed to obtain a positive PCR product (about 1017-bp) using single-cross primers (Supplementary Fig. S6) indicating that, the insertion of *tetM* at the *hlyC* site is due to a double cross-over event.

### Loss of hemolytic phenotype in the mutants

**Figure 8:**
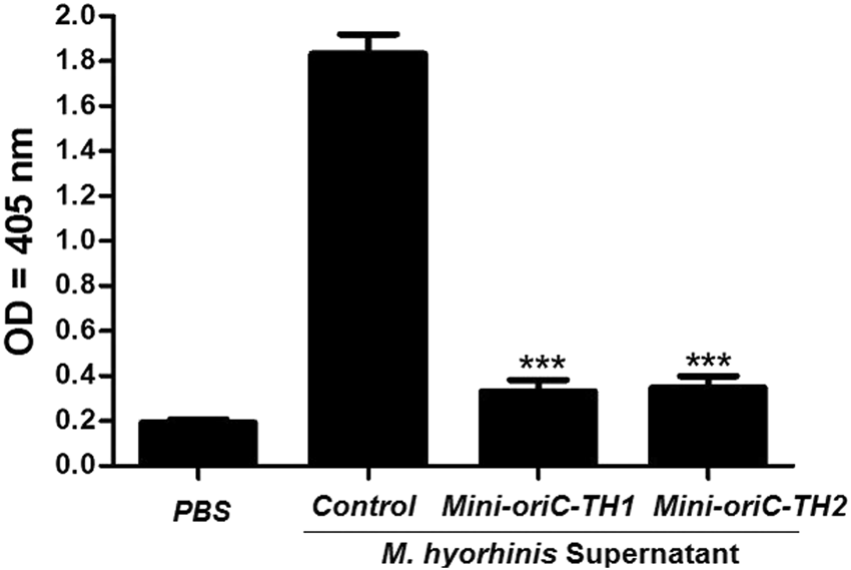
Hemolytic activity of the wild-type and *hlyC* mutants of *M*. *hyorhinis*. The supernatant of wild-type and *hlyC* mutants of *M*. *hyorhinis* were collected and incubated with mouse RBCs. PBS was used as a negative control. The released hemoglobin was determined by measuring the OD at 405 nm. All reactions were performed in triplicate. Data are presented as Means ± SD (n = 3). *P* < *0*.*001* versus control.

In *M*. *hyorhinis*, disruption of *hlyC* was expected to diminish its ability to lyse mouse RBCs. The wild-type and the mutant cells grown in KM2 medium and the supernatant were used to assay the hemolytic activity. Mouse RBCs were incubated with the supernatant while the control with PBS. The hemolytic activity was measured at 405 nm. We found that, the supernatant collected from the mutant colonies significantly (*P* < *0*.*001*) lacks the ability to lyse mouse RBS compared to wild-type bacterial supernatants (Fig. 8). Therefore, this is a first study demonstrated the gene associated with a hemolytic phenotype in mycoplasmas

## Discussion

In this study, we have described the construction and the use of a series of *M*. *hyorhinis oriC*-plasmids. Although the transformation of *M*. *hyorhinis* has been reported earlier with polyethylene glycol (PEG)^5^, the usage of the host-vector systems for genetic studies of *M*. *hyorhinis* has not so far been implemented. Therefore, a host-vector system which can be utilized for *M*. *hyorhinis* genetic studies is a prerequisite. The electro-transformation conditions optimized in this study are suitable for *M*. *hyorhinis*. However, some factors such as the use of minimum inhibitory concentration of tetracycline, high voltage and the excess amount of plasmid DNA should be considered.

*OriC*-plasmids have been successfully used in a number of Mollicutes^15, 16, 18, 19^. Here, the origin of replication region of *M*. *hyorhinis* genome has been identified and was used to construct *oriC*-plasmids for this bacterium for the first time. The prediction of large *oriC* (L*oriC*) region of *M*. *hyorhinis* chromosome has identified two dnaA boxes upstream and downstream the *dnaA*, and this L*oriC* was proved to be functional in the *pGEMT-LoriC* plasmid. In the *M*. *hyorhinis* transformants, the *pGEMT-LoriC* plasmid was found to replicate as a free extra-chromosomally before integrating into the host chromosome during passaging, similarly to the *pBOT1* plasmid in *Spiroplasma citri* ^26^.

The length of the *oriC* sequences has been shown to influence the recombination efficiency. Furthermore, the presence of a complete *dnaA* is not essential to driving the replication in *oriC*-plasmids as in *Spiroplasma citri* ^13, 19^. Therefore, a plasmid containing a minimum *oriC* fragment was constructed to decrease the frequency of plasmid integration into the *oriC*-region of *M*. *hyorhinis* ^15^. Indeed, free extra-chromosomal *oriC*-plasmids were obtained when the *pGEMT-MoriC* plasmid containing a minimum *oriC*-fragment (876-bp) used, similar to previous observations^14, 19^.

Tetracycline was chosen as the selection marker as it has been used in multiple mycoplasma species and that *M*. *hyorhinis* strain HUB-1 stored in our laboratory is tetracycline sensitive^27^. The *oriC*-based plasmids described here have several useful features that will provide flexibility in future genetic studies. The multiple cloning sites located upstream and downstream of *tetM* and the spiralin gene promoter make these *oriC*-plasmids compatible for the subsequent cloning of foreign DNA (such as *GFP* or other antigens) for many applications in *M*. *hyorhinis*. Indeed, this may encourage *in vivo* imaging following *M*. *hyorhinis* infection or even development of recombinantly engineered vaccines when foreign antigens are expressed.

The hemolytic activity has been previously described in some mycoplasmas, however, there has been no previous study showing that a particular gene has hemolytic activity in mycoplasmas. In this study, we investigated the function of *hlyC* by using our *oriC*-plasmids, and the data show that, the *hlyC* is responsible for the hemolytic activity observed in *M*. *hyorhinis*. Analysis of the *hlyC* DNA sequence present in different strains of *M*. *hyorhinis* (**HUB-1** Accession CP002170 Region: 304679..305920, **DBS 1050** Accession CP006849 Region: 438049..439291, **GDL-1** Accession CP003231 Region: 438066..439308, **MCLD** Accession CP002669 Region: 170809..172050, **MDBK-IPV** Accession CP016817 Region: 437951..439192 and **SK76** Accession CP003914.1 Region:305879..307120) by the blast and Clustal V methods, has revealed only one base different (in **DBS 1050** and **GDL-1** strains) in the *hlyC* sequence (Supplemental Fig. S7). The conservancy of *hlyC* sequence in these strains may indicate its potential in the pathogenesis of these strains although of the fact that the strains of *M*. *hyorhinis* express differences in virulence^28^. However, the association between the *hlyC* and virulence of *M*. *hyorhinis* remained to be evaluated. Due to the available genome sequences of *M*. *hyorhinis* and the importance of this bacterium in the development of arthritis as well as cancers *in vitro*, the development of *oriC*-plasmids for *M*. *hyorhinis*, could allow identification of virulence factors, and understand its pathogenesis. In conclusion, our study has developed useful *oriC*-plasmids for *M*. *hyorhinis* and also identified the association between *hlyC* and the hemolysis phenotype for the first time.

## Material and Methods

### Bacterial strains and cultural conditions

The *M*. *hyorhinis* strain HUB-1 (GenBank accession CP002170.1) was kindly provided by Professor Xiao Shaobo (Huazhong Agricultural University, Wuhan, China) and cultured in a modified Friis medium (KM2 medium) containing 20% (v/v) swine serum^29^ at 37 °C. The solid medium (KM2-Agar) was prepared by adding 0.7% Agar (Biowest Agarose ^®^ G-10; Gene Company Limited, Chi Wan, Hong Kong) to the liquid KM2 medium and was incubated at 37 °C until the growth of the visible colonies. For the growth of mutants, tetracycline hydrochloride (Sigma-Aldrich) was added at 5.0 μg/ml and 2.5 µg/ml to the solid and liquid media respectively.

### Construction of *oriC*-plasmids

We used *pGEM®-T* vector to construct the *oriC*-plasmids. Images of PCR ethidium bromide-stained agarose gels were acquired and with Gel Doc™ XR + System with Image Lab™ Software (Bio-Rad, USA). The tetracycline gene, *tetM* (ID: AGI19285.1) was PCR amplified (using **P1** primers, Table 1) from *pSE-1* vector provided by Professor Xiao Shaobo (Huazhong Agricultural University, Wuhan, China) and cloned into *pGEM®-T* vector at *Spe*I/*Pst*I restriction enzymes sites by infusion cloning methods to generate *pGEMT-tetM* plasmid. Since common bacterial cloning vectors such as *pGEM®-T* vector or genes from *E*. *coli* cannot express in the *M*. *hyorhinis* from their own promoter, the spiralin gene promoter (from *Spiroplasma citri*, GI: 2384684, amplified from *pSE-1* vector using **P2** primers, Table 1) was cloned upstream of *tetM* at *Spe*I restriction enzyme site to generate *pGEMT-Sp/tetM* plasmid. The large fragment of the origin of replication (L*oriC*) of *M*. *hyorinis* was identified (Fig. 1), PCR amplified from of *M*. *hyorinis* genomic DNA using **P3** primers, Table 1 and cloned at *Apa*I restriction enzyme site to generate *pGEMT-LoriC* plasmid.

To amplify a minimum *oriC* fragment (M*oriC*), the flanking regions of *dnaA* that contains the AT-rich, were PCR amplified with **P4A** primers, Table 1 as fragment A (upstream *dnaA*, 563-bp) and with **P4B** primers, Table 1 as fragment B (downstream *dnaA*, 313-bp). These two fragments were joined by overlapping PCR (using **P4A-F** and **P4B-R** primers, Table 1) to form a single fragment of 876-bp as minimum *oriC* (Fig. 1). This minimum *oriC* PCR product was cloned into *pGEMT-Sp/tetM* at *Apa*I restriction enzyme site to generate the *pGEMT-MoriC* plasmid as a minimum *oriC*-plasmid. The design of the fragments A and B were shown in the diagram presented in (Fig. 1), while the primers in (Table 1).

We chose to disrupt *hlyC* (NCBI Sequence: WP_o14582557.1) because we hypothesized that if *hlyC* confers hemolytic activity, it may be easy to measure the hemolytic phenotype. To construct plasmid targets *hlyC*, both hemolysin left and right arms were at least 450-bp to provide adequate homologous sequences to facilitate recombination at the target gene^10, 11, 22^. These arms were PCR amplified and cloned to flank *tetM* as well as the spiralin gene promoter since we aimed to knock-in a functional *tetM* at the hemolysin site. The left arm (LA) of *hlyC* (amplified from *M*. *hyorhinis* DNA using **P5** primers, Table 1) was cloned into *pGEMT-MoriC* plasmid upstream spiralin gene promoter at *Sac*II restriction enzyme site while right arm (RA) of *hlyC* (amplified from *M*. *hyorhinis* DNA using **P6** primers, Table 1) cloned downstream of *tetM* at *Sal*I restriction enzyme site. The resultant plasmid named mini-*oriC* targeting plasmid-1 (*Mini-oriC-HT1*) and was used to inactivate *hlyC* by homologous recombination.

The recombinase gene (*recA*) of *E*. *coli* was found to enhance the homologues recombination in *M*. *mycoides* subsp. *capri* ^7^. Therefore, the *recA* (GI:446885887) was PCR amplified from *E*. *coli* BL-21 DNA using **P7** primers, Table 1. At the same time, the spiralin gene promoter was PCR amplified with **P8** primers, Table 1 and joined by overlapping PCR with *recA* (using **P8-F** and **P7-R** primers, Table 1) to form a single fragment which was cloned into *Mini-oriC-T1* plasmid at *Nco*I restriction enzyme site. The resulted plasmid named mini-*oriC* targeting plasmid-2 (*Mini-oriC-HT2*) and was also evaluated for its functionality to inactivate *hlyC* by homologous recombination. All cloning steps were verified by colony PCR and analysis of DNA sequencing. The diagram of the vectors constructions was shown in (Fig. 3), while the primers used to amplify or join the PCR fragments into each, were listed in (Table 1).

### Electroporation of *M*. *hyorhinis*

*M*. *hyorhinis* were used at approximately 10E8 CCU. Electroporation of *M*. *hyorhinis* was carried out by a procedure similar to previously described methods with minor modifications^19, 27^. Briefly, the culture centrifuged at 6,000 rpm for 15 min at 4 °C, and the pellet washed three times with ice-cold electroporation buffer (272 mM Sucrose, 200 mM HEPES, pH 7.2) and resuspended in 100 µl ice-cold electroporation buffer. Aliquot of 0.1 ml of competent cells was mixed with 20 µg plasmid DNA. The mixture was then placed in a pre-chilled sterile electroporation cuvette (2 mm electrode gap, Bio-Rad Laboratories, Hercules, CA) and pulsed immediately with ECM 630 Electroporation System (Harvard Apparatus BTX, Holliston, MA, USA) at 2.5 KV, 125 Ω, and 25 µF. The mixture was immediately diluted with 900 µl of cold KM2 broth, incubated on ice for 5 min, and recovered at 37 °C for 3 hours. The cells were diluted 1:10 and selected on KM2-Agar containing 2.5 µg/ml tetracycline and grown at 37 °C till colonies appeared. Tetracycline-resistant colonies were sub-cultured in 2 ml of KM2-broth containing 5.0 μg/ml tetracycline. Transformants were passaged by inoculating 100 μl of culture into 900 μl of broth containing 5.0 μg/ml of tetracycline.

### Screening of the plasmids by PCR

Following transformation with *pGEMT-LoriC* and *pGEMT-MoriC* plasmids, the cells were diluted and plated on KM-Agar containing 2.5 µg/ml tetracycline. Individual colonies of *M*. *hyorhinis* were sub-cultured in KM2 medium containing 5.0 µg/ml tetracycline at 37 °C several times to eliminate the residual plasmid from the medium. To detect the presence of the plasmids in the transformants, total DNA was extracted from 2 ml *M*. *hyorhinis* cultures using a genomic DNA purification kit (TIANGEN, Beijing, China). We performed a PCR amplifying a fragment of the *tetM* (part of the *oriC*-plasmid backbone) using universal *tetM* screening primers (**P9**, Table 1)^30^. This PCR resulted in 339-bp *tetM*-product in the presence of free or integrated *oriC* plasmids.

### Analysis of hemolytic phenotype

A single clone of the mutant and wild-type *M*. *hyorhinis* was grown in KM2 medium containing 5.0 µg/ml tetracycline and the culture centrifuged at 10,000× g for 10 min at 4 °C. The supernatants were carefully collected and analyzed for the hemolytic activity using fresh mouse RBCs collected from healthy mice. The hemolytic activity was determined as previously described^31^ with the minor modifications. Briefly, mouse RBCs were incubated with the supernatant collected from the mutant and wild-type *M*. *hyorhinis* for 2 hours at 37 °C. Control samples were incubated with PBS. The samples were centrifuged at 1,500× g for 10 min and the released hemoglobin was determined by measuring the OD at 405 nm. All reactions were performed in triplicate.

### Statistical analysis

Data obtained from three individual experiments were recorded as Mean ± SD and subjected to one-way analysis of variance (ANOVA) using SPSS (version 16.0, SPSS Inc., Chicago, IL, USA). *P* < *0*.*05* was considered statistically significant.

## Electronic supplementary material

Supplementary Information
