# Supplementary material for: Development of oriC-plasmids for use in Mycoplasma hyorhinis
Source: Sci Rep. 2017 Sep 6;7:10596. doi: 10.1038/s41598-017-10519-3 (PMC5587638; doi:10.1038/s41598-017-10519-3)

Manuscript Title: Development of oriC-plasmids for use in *Mycoplasma hyorhinis*

Journal: Scientific Reports

Hassan Z. A. Ishag[1, 2], Qiyan Xiong[1]*, Maojun Liu[1], Zhixin Feng[1] and Guoqing Shao[1]

[1]Institute of Veterinary Medicine, Jiangsu Academy of Agricultural Sciences, Key Laboratory of Veterinary Biological Engineering and Technology, Ministry of Agriculture, National Research Center for Engineering and Technology of Veterinary Bio-products, Nanjing 210014, China

[2] College of Veterinary Sciences, University of Nyala, Nyala, Sudan

*Corresponding author: Qiyan Xiong

E-mail: qiyanxiongnj@163.com

Phone: +86 25 84390880

Fax: 86-25-84391973

Electronic Supplementary File: Figures and Legends

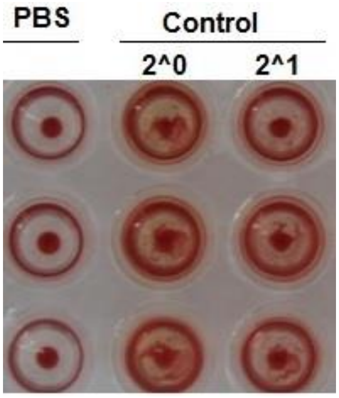

Supplemental Fig.S1 Hemolytic activity of *M. hyorhinis*. Mouse RBCs was incubated with supernatant of wild-type *M. hyorhinis* (2-fold dilution) and PBS (control). The hemolytic activity was observed. The image was cropped and full-length image is included in the **Supplementary Fig.S11**.

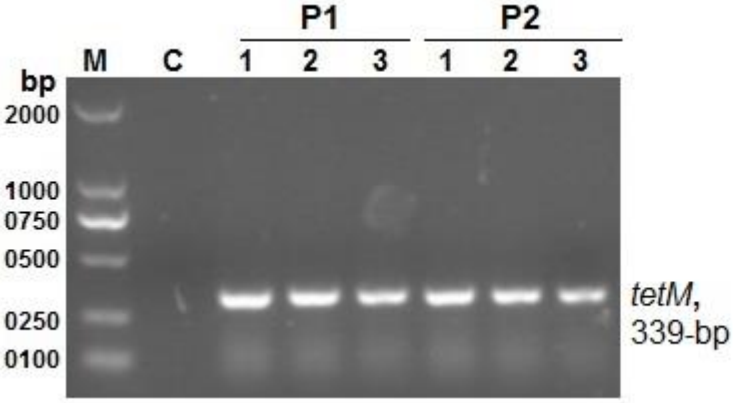

Supplemental Fig.S2 Detection of the *pGEMT-LoriC* and *pGEMT-MoriC* plasmids in a single clone sub-culture passages. DNA was extracted on the third passages from control untransformed culture (C), cells transformed with the *pGEMT-LoriC* plasmid (P1) and cells transformed with the *pGEMT-MoriC* plasmid (P2). The presence of these plasmids in the transformants was detected by *tetM*-specific PCR (using **P9** primers, Table-1) amplifying an about 339-bp of *tetM*. The gel image was cropped and full-length gel is included in the **Supplementary Fig.S12**.

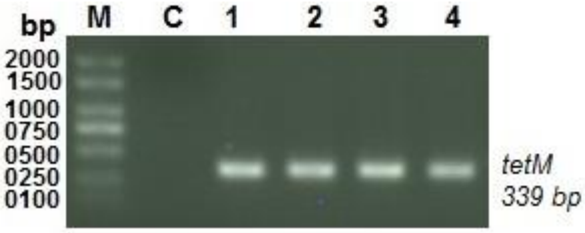

Supplemental Fig.S3 Detection of the *pGEMT-LoriC* plasmid following integration by single cross over at the *oriC* site of *M. hyorhinis.* Analysis of the DNA extracted from *pGEMT-LoriC* transformants by *tetM* specific PCR (using **P9** primers, Table-1), had detected the *tetM* (about 339-bp) and further confirmed the presence of the integrated *pGEMT-LoriC* plasmid. M = DNA molecular marker, C = control untransformed culture and the lanes (1-4) represent number of tetracycline resistant colonies tested. The gel image was cropped and full-length gels and blots are included in the **Supplementary Fig.S13**.

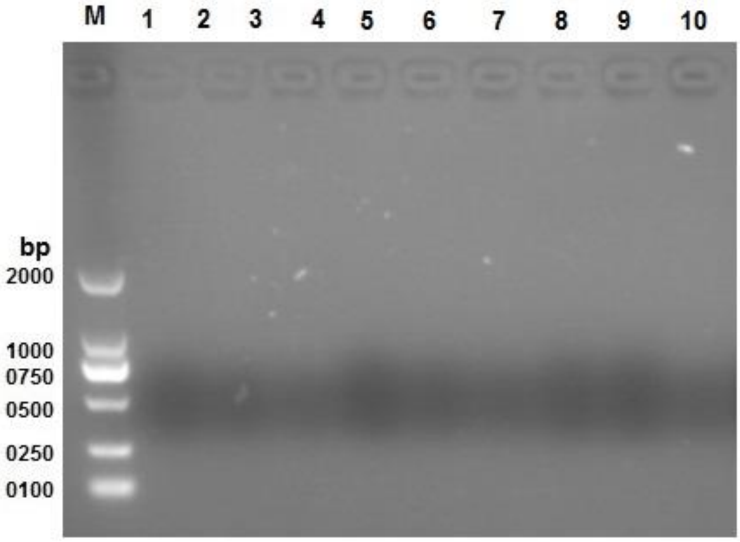

Supplemental Fig.S4. Investigation of the possible integration of *pGEMT-MoriC* plasmid containing Mini-*oriC* (M*oriC*) at the *oriC*-region of *M. hyorhinis.* Following integration of the plasmid at the *oriC* region, a predicted 2122-bp PCR product could be detected with specific integration primers (**P10**, Table-1). We got no PCR product indicating the absence of plasmid integration at this region by single cross-over. Lanes (1-10) indicate number of tetracycline resistant colonies investigated. The gel image was cropped and full-length gel image was included in the **Supplementary Fig.S14**.

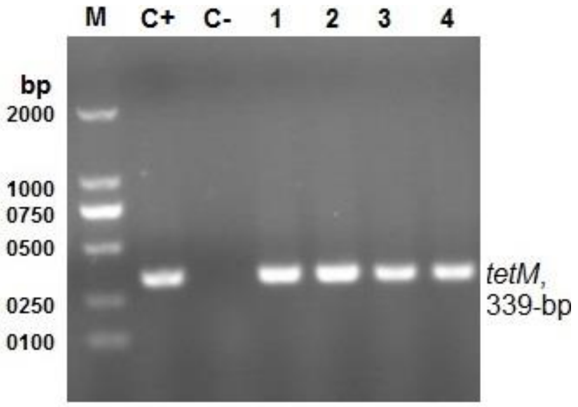

Supplemental Fig.S5 Detection of the *tetM* insertion at the hemolysin site using *Mini-oriC-HT2* plasmid. The presence of *tetM* was investigated with *tetM* specific PCR (using **P9** primers, Table-1 and the 3072-bp PCR product that was amplified with *hlyC* flanking primers **P11**, Table-1 as a template). M = DNA molecular marker, C+ = *Mini-oriC-HT2* positive control *(*untransformed plasmid), C = negative control (untransformed culture) and the lanes (1-4) represent number of colonies tested. The gel image was cropped and full-length gels and blots are included in the **Supplementary Fig.S14**.

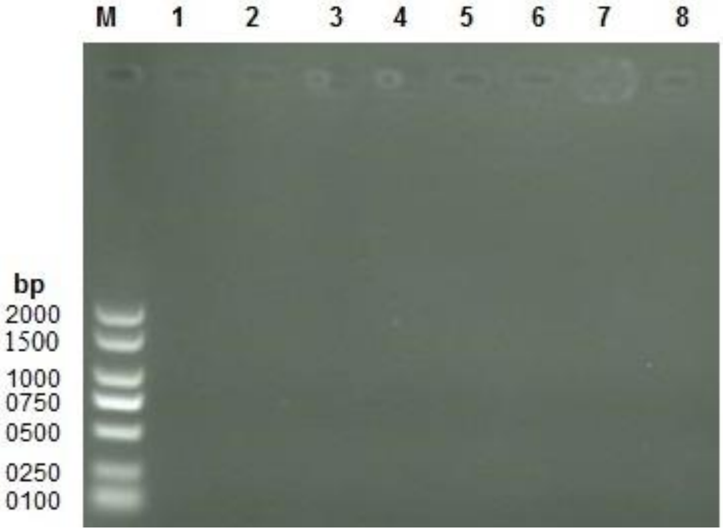

Supplemental Fig.S6 Investigation of a single cross-over event between any arm of *hlyC* leading to integration of the full *Mini-oriC-HT1* plasmid. The PCR using single-cross primer-F (**P12-F**) and single-cross primer-R (**P12-R**) to detect the predicted product following integration of full *Mini-oriC-HT1* plasmid at the right arm of *hlyC* by single cross-over, had failed to detect a band of about 1017-bp, indicating the insertion of *tetM* at the hemolysin site is due to a double cross-over event. Lanes (1-8) indicate number of tetracycline resistant colonies investigated. The gel image was cropped and full-length gel is included in the **Supplementary Fig.S15**.

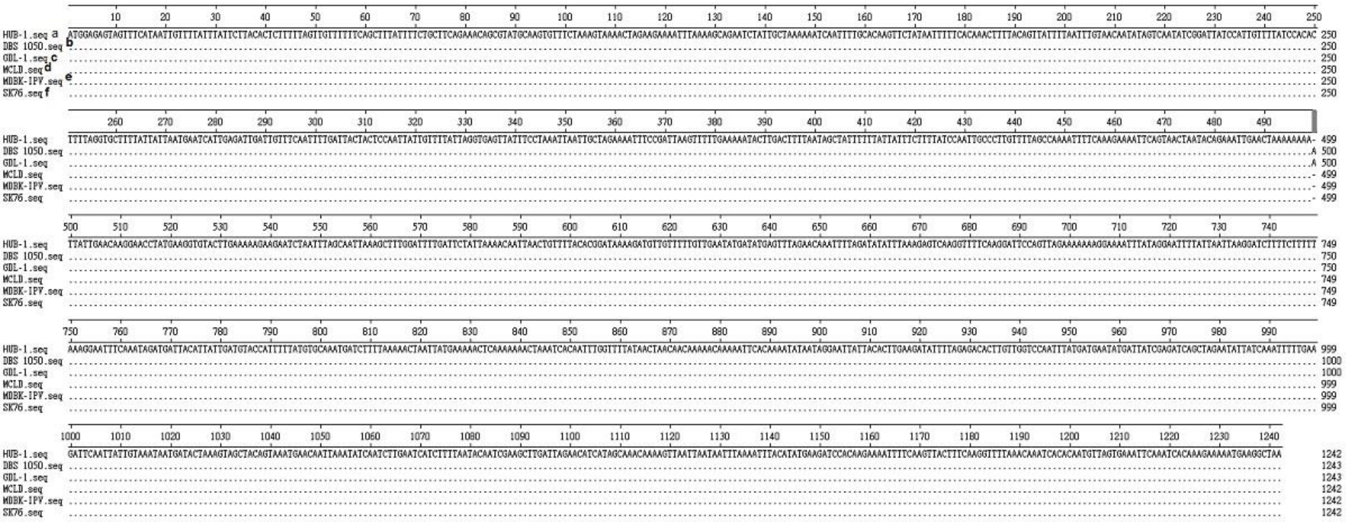

Supplemental Fig.S7 Alignment of the DNA sequences of *hlyC* in different *M .hyorhinis* strains. The alignment of the DNA sequences of *hlyC* of the six *M .hyorhinis* strains: [a]= **HUB-1** (Accession CP002170 Region: 304679...305920), [b]= **DBS 1050** (Accession CP006849 Region: 438049..439291), [c]= **GDL-1** (Accession CP003231 Region: 438066..439308), [d]= **MCLD** (Accession CP002669 Region: 170809..172050), [e]= **MDBK-IPV** (Accession CP016817 Region: 437951..439192) and [f]= **SK76** (Accession CP003914.1 Region:305879-307120) by Clustal V method showed that only one base different in *hlyC* sequence in two strains (DBS 1050 and GDL-1).

Supplementary Fig.S8. Full-length gels images of Fig.2

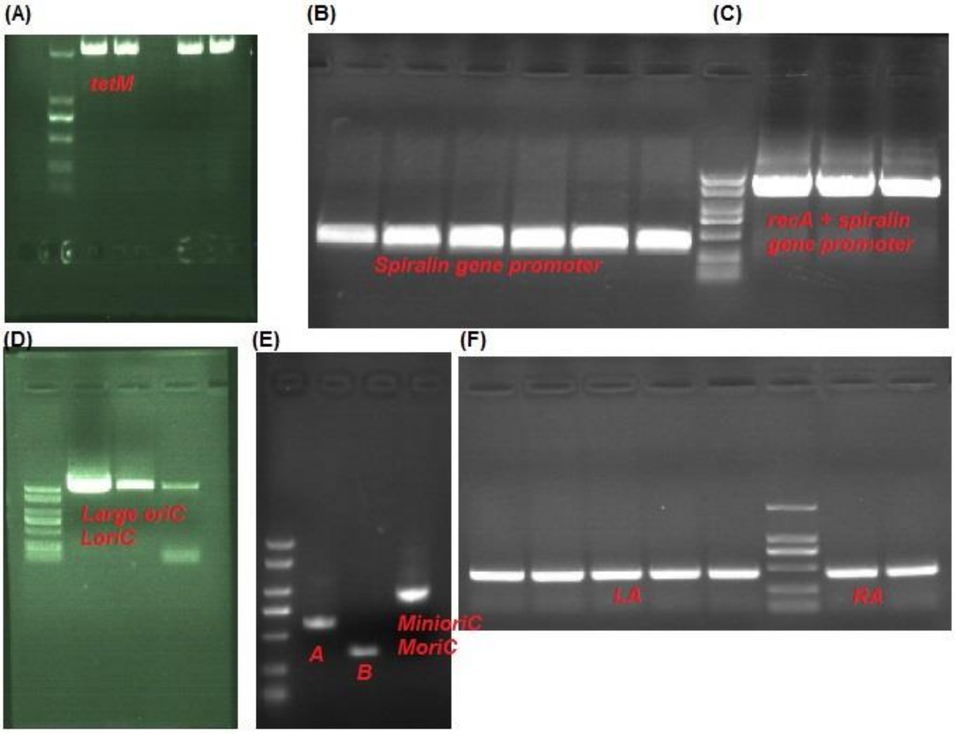

Supplementary Fig.S9. Full-length gel image of Fig.4

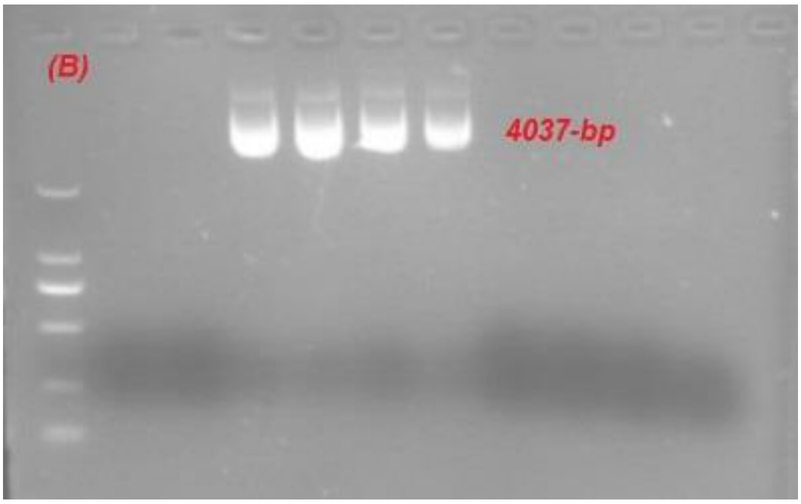

Supplementary Fig.S10. Full-length gel image of Fig.6

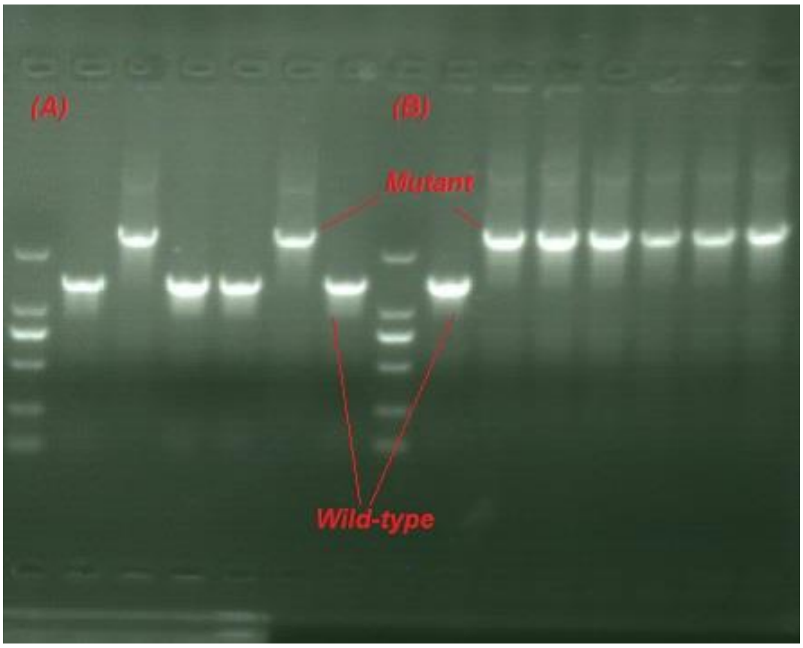

Supplementary Fig.S11. Full-length image of Fig.S1

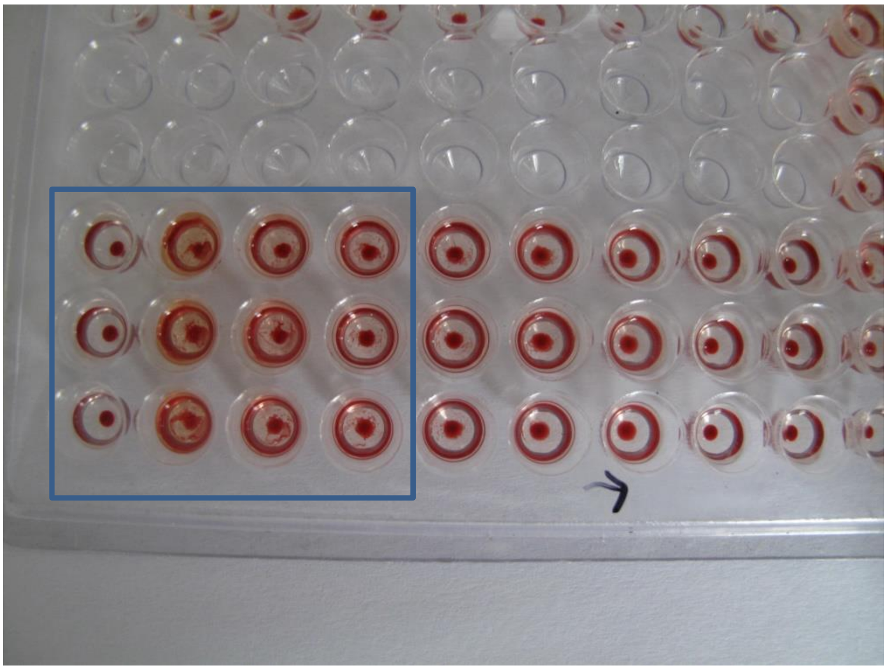

Supplementary Fig.S12. Full-length image of Fig.S2

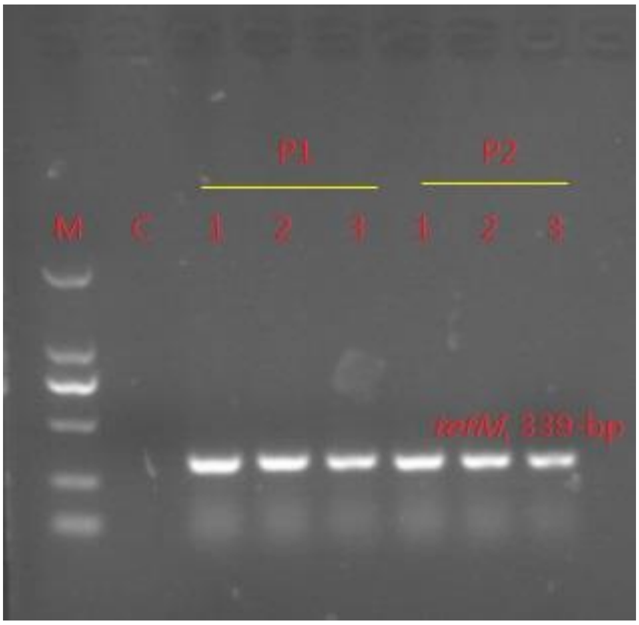

Supplementary Fig.S13. Full-length image of Fig.S3

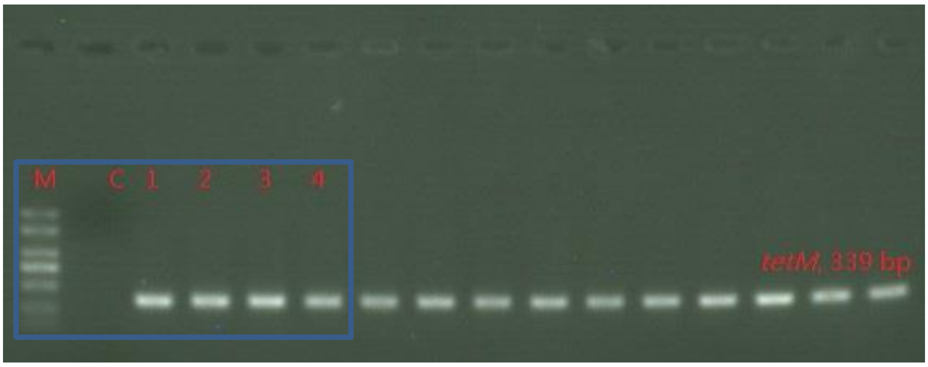

Supplementary Fig.S14. Full-length gel image of Fig.S4

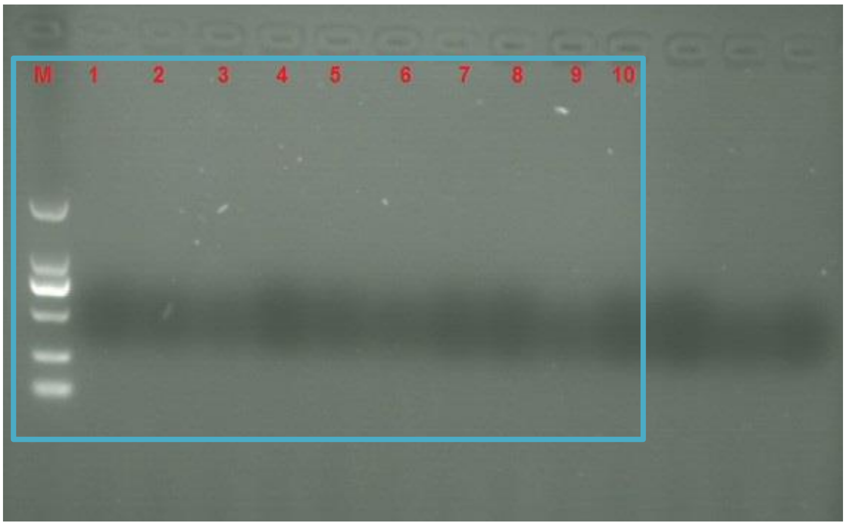

Supplementary Fig.S15. Full-length gel image of Fig.S5

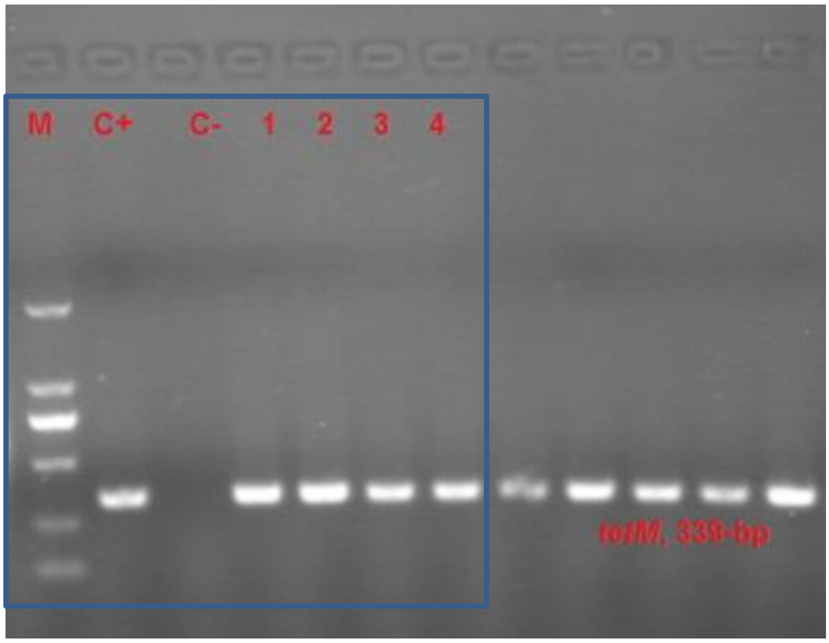

Supplementary Fig.S16. Full-length gel image of Fig.S6

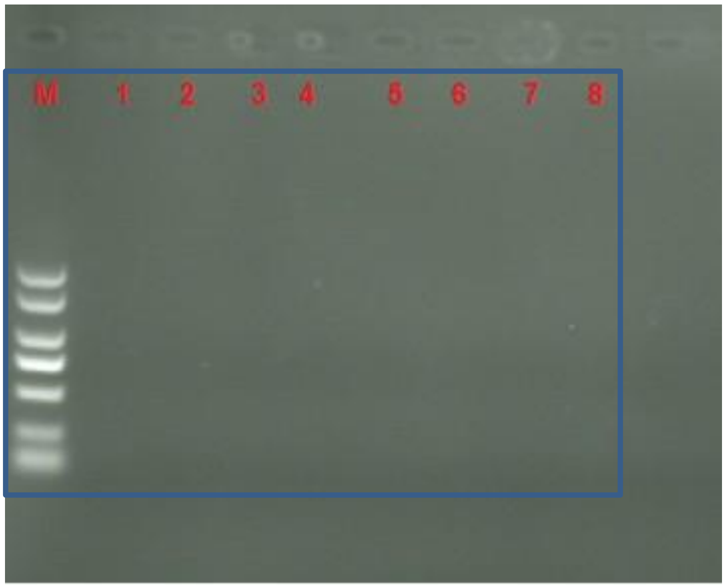

Supplement: Supplementary file 1 — Supplementary Information [file 41598_2017_10519_MOESM1_ESM.pdf]
